# Clinical Manifestations of Wegener Granulomatosis in Iranian Ethnicities Using the K-Means Algorithm: A Descriptive Study

**DOI:** 10.1055/s-0043-1767803

**Published:** 2024-02-05

**Authors:** Fahimeh Khedmatkon, Samira Alesaeidi, Fatemeh Hajialiasgari, Jaleh Shoshtarian Malak

**Affiliations:** 1 Department of Academics, Tehran University of Medical Sciences, Tehran, The Islamic Republic of Iran; 2 Department of Internal Medicine, School of Medicine, Shariati Hospital, Tehran University of Medical Sciences, Tehran, the Islamic Republic of Iran

**Keywords:** Wegener granulomatosis, granulomatosis with polyangiitis, ethnicity, data mining, clustering

## Abstract

**Introduction** Wegener granulomatosis (WG) appears with clinical symptoms, including recurrent respiratory infection, renal manifestations, and nonspecific systemic symptoms.

**Objective** To study the clinical manifestations of WG in Iranian ethnicities, and data on 164 patients were recorded from 2013 to 2018.

**Methods** The data included demographics, symptoms, and the Birmingham Vasculitis Activity Score (BVAS). The symptoms involved the following sites: the nose, sinus, glottis, ears, lungs, kidneys, eyes, central nervous system, mucous membranes, skin, heart, stomach, intestine, as well as general symptoms. The clinical manifestations of nine ethnicities were analyzed.

**Results** In total, 48% of the patients were male and 51% were female, with a median age of 51 years. The BVAS was of 15.4, the sites most involved were the sinus ( *n* = 155), nose ( *n* = 126), lungs ( *n* = 125), and ears ( *n* = 107). Gastrointestinal ( *n* = 14) and cardiac ( *n* = 7) involvement were less common. Among the patients, 48.17% were Persian, 13.41% were Azari, 11.17% were Gilaki, 11.17% were Kurd, and 10.9% were Lor.

**Conclusion** Our findings indicated that the sinus, nose, lungs, and ears were the sites most involved, and gastrointestinal and cardiac involvement were less common. In the present study, involvement of the upper and lower respiratory tract was higher than that reported in Western and Asian case series. Moreover, we report for the first time that, in all patients with ear involvement, the left ear was the first to be affected. The clinical manifestations among Iranian ethnicities were not different, and the Gilaki ethnicity had the highest BVAS, mostly because the weather was humid; therefore, in Iran, in areas with humidity, the rate of the disease was higher.

## Introduction

Wegener granulomatosis (WG), or granulomatosis with polyangiitis (GPA), is a systemic vasculitis that affects the upper and lower respiratory tract, the kidneys, the joints, the eyes, the skin, the nervous system, and the heart. 1 It appears with clinical symptoms that include recurrent respiratory infection, renal manifestations, and nonspecific systemic symptoms. 2

Epidemiological studies on antineutrophilic cytoplasmic antibody (ANCA)-associated vasculitis (AAV) have resulted in the development of classification criteria and disease definitions. In general, studies show that the incidence is of 10 to 20 people per million people per year; men are more likely to get the disease than women, in a ratio of 1.5 to 1, and more old people between the ages of 65 and 74 are affected by the disease (6 cases/100 thousand). 3 The overall prevalence of GPA in European populations has been estimated to range from 2 to 38 cases per million. 4 in Japan, the prevalence is of 17.8 people per million per year, and women are more likely to get the disease than men. 5

Previous studies 6 show that, throughout the past 15 years, the epidemiology of GPA has become better understood, and they estimate that the prevalence in European countries ranges from 24 to 157 people per million, and the annual incidence rates range from 3 to 14 people per million.

In the present study, the role of ethnicity and gender in the course of WG was investigated. We aimed to describe the clinical manifestations and outcomes of WG in Iranian ethnicities.

## Materials and Methods

### Data Source and Study Population

The data of 164 patients referred to the hospital where the study was conducted from 2013 to 2018 were recorded in a database developed using the Microsoft Excel (Microsoft Corp., Redmond, WA, United States) software. The data included demographics (gender, year of birth, and ethnicity), symptoms, and the severity of the condition according to the Birmingham Vasculitis Activity Score (BVAS). Due to the wide range of symptoms of GPA, we used the BVAS, in which the types of symptoms and the organs involved are classified, to record the symptoms of the patients, which may affect the following sites: the nose, sinus, glottis, ears, lungs, kidneys, eyes, central nervous system, , mucous membranes, skin, heart, stomach, intestine, as well as general symptoms.

**Fig. 1:**
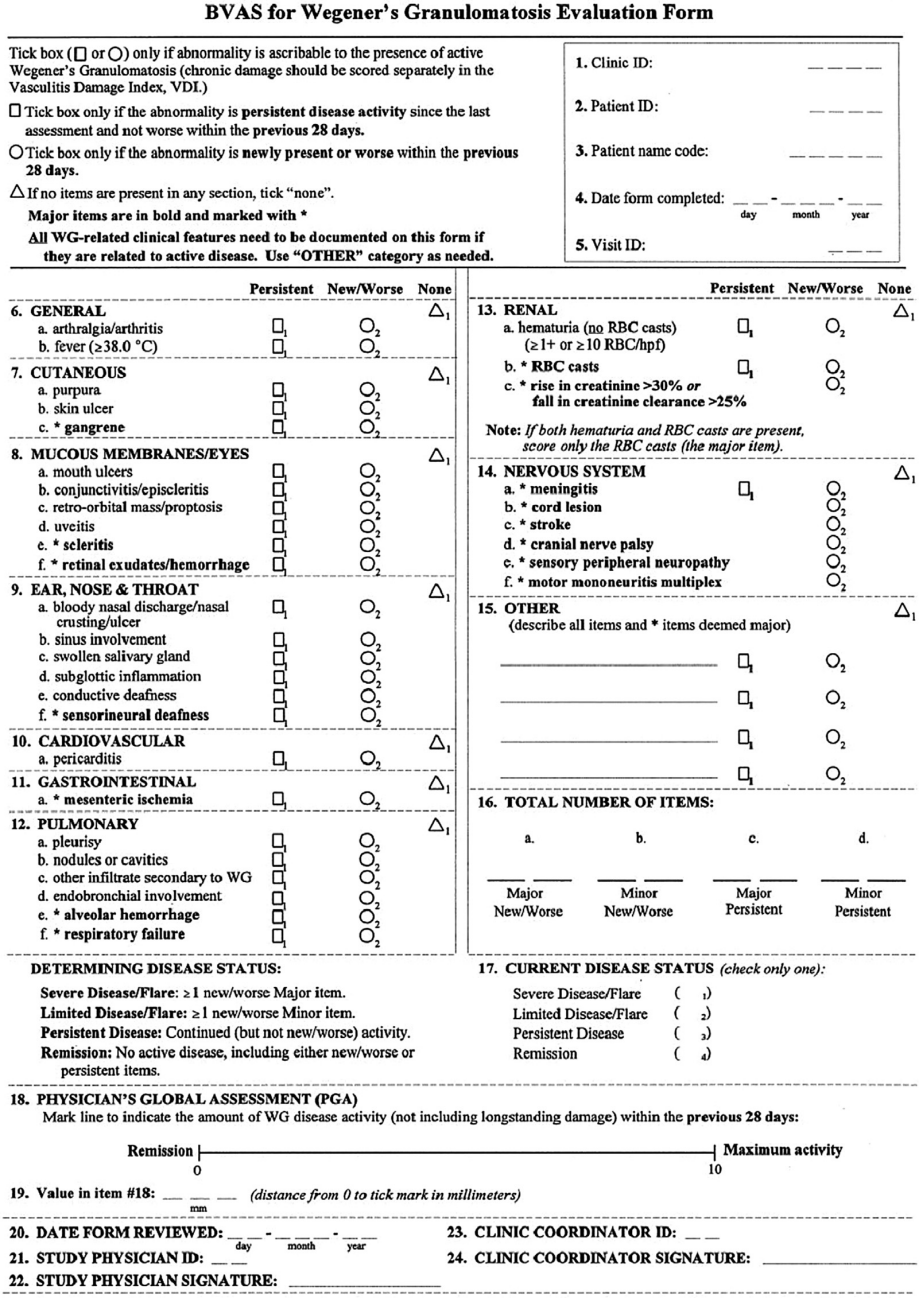
Evaluation form of the Birmingham Vasculitis Activity Score for Wegener Granulomatosis (BVAS/WG).

To assess the condition, we used a WG-specific disease activity index based on the BVAS (BVAS/WG), which was applied as an evaluation form ( Fig. 1 ). 7

### Variables

In the present study, the gender, age, ethnicity, clinical symptoms, and geographical location of the patients were identified in the Excel spreadsheet. Ethnicity was classified as Persian, Azari, Kurd, Lor, Arab, Gilaki, Baloch, Bakhtiari, and Turkman. Table 1 shows the percentages of the ethnic groups that comprise the population of Iran, and Persian, Azari, and Kurd are the groups with the highest percentages. ^8–10^ The province and city of the patient's residence as well as ethnicity were extracted based on the first three digits of the patient's national code.

**Table 1:** Ethnic groups of the study sample

Ethnic groups	Total
Persian	54%
Azari	16%
Kurd	10%
Lur	5%
Gilaki	4%
Turkman	2%
Arab	2%
Balouch	2%
Bakhtiari	1%

As for the clinical symptoms and the sites of involvement, nasal involvement may result in rhinitis, nasal ulcer, epistaxis or nosebleeds, peroration of the nasal septum, saddle nose, and polyps. Sinus involvement may cause sinusitis, mastoid erosion, and sclerosis. Glottis involvement may result in subglottis and plegia of the glottis. Ear involvement may cause conductive hearing loss and sensorineural hearing loss. Lung involvement may result in asthma, wheezing, hemoptysis, infiltration, ground-glass opacity, alveolar hemorrhage, nodule or cavitation, effusion, subsegmental atelectasis, bronchiolitis, mosaic pattern, bronchiectasis, endobronchial involvement, interstitial lung disease (ILD), and infarction. Renal involvement may cause kidney protein, blood, RBC > 10, kidney casts, 1.4 < CR < 2.8 and CR > 2.8. Eye involvement may result in episcleritis, scleritis, dacryocystitis, proptosis, ptosis, cellulitis, blurred vision, visual loss, conjunctivitis, vasculitis, keratitis, and uveitis. Central nervous system involvement may cause headache, 10th, 7 th nerve, 6th nerve, 5th nerve, 4th nerve, 3rd nerve, 2nd nerve, 9, 10, 11 (jugular fossa), 12th, 1st, LOC, seizure, vasculitis, thrombosis, osteomyelitis, mononeuritis multiplex, meningitis, and encephalitis. And the general symptoms may be fever, night sweats, arthralgia, myalgia, weight loss, and arthritis. Mucous membrane involvement may cause oral mucosa ulcer, mucous gang/erosion, mucous salivary, and genital mucosa ulcer. Skin involvement may result in vasculitis and ulcers. Cardiac involvement may cause heart effusion, heart failure, and thrombophlebitis. Gastrointestinal involvement may result in, ischemia, pancreas, splenomegaly, hepatomegaly, hepatitis, and VDI.

### Clustering Model

Data mining is used to discover hidden relationships and patterns. 10 K-means is a clustering method used for segmentation. The initialization procedure chooses the initial cluster center randomly from input data. While performing the data segmentation, the clusters are generated. 11

## Results

### Association of the BVAS with Clinical Symptoms

Data mining regarding the WG patients using the K-means method used attributes such as BVAS, nose, sinus, glottis, ear, lung, renal, eye, central nervous system, general, mucous membranes, skin, heart, and gastrointestinal. The data used are WG patients based on clinical symptoms (2013–2018) originating from the Hospital. The data were processed in two clusters: high BVAS (cluster 0) and normal BVAS (cluster 1). The initial centroid value is determined by the largest value (cluster 0), and the average value (cluster 1) is based on the attributes.

In the clustering of the data of the WG patients, used the k-means algorithm to cluster the data, as shown in Table 2 . Iteration in the process of execution on k-means to cluster the databased on the cluster center of distance value.

**Table 2:** Centroid Initial Data

Attribute	Cluster_0	Cluster_1
Birmingham Vasculitis Activity Score	22.986	10.411
Nose	0.783	0.916
Sinus	0.986	0.758
Glottis	0.116	0.663
Ear	0.652	0.653
Lung	0.899	0.263
Kidney	0.681	0.2
Eye	0.493	0.2
Central nervous system	0.507	0.179
General	0.478	0.158
Mucous membrane	0.203	0.126
Skin	0.232	0.084
Heart	0.087	0.053
Stomach and intestine	0.087	0.011

**Fig. 2:**
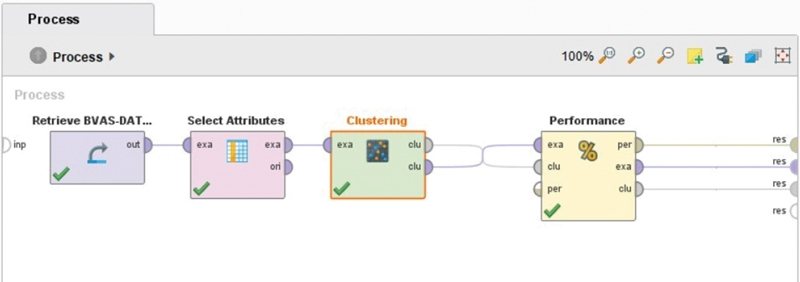
Design of K-means algorithm with k = 2.

**Fig. 3:**
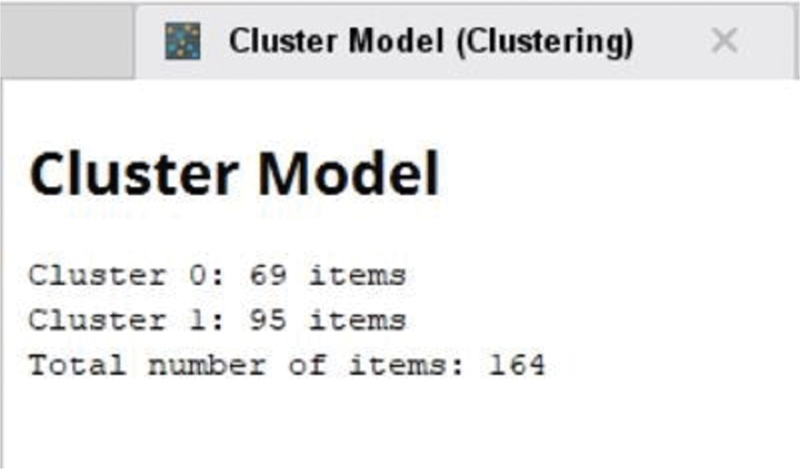
Result of the clustering.

**Fig. 4:**
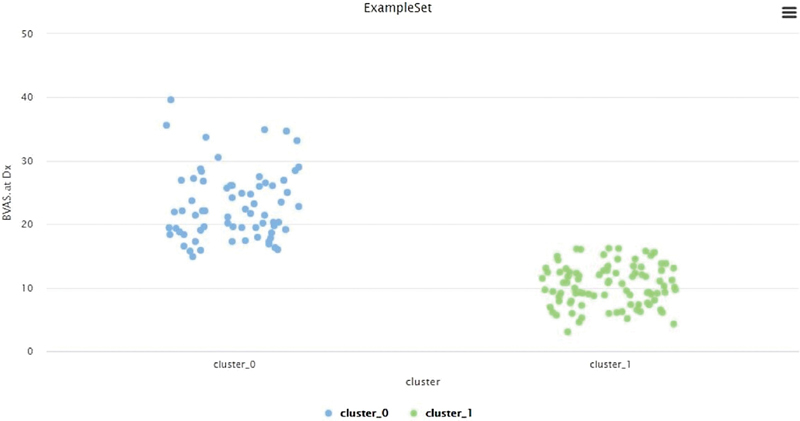
Result of the k-means with RapidMiner.

**Fig. 5:**
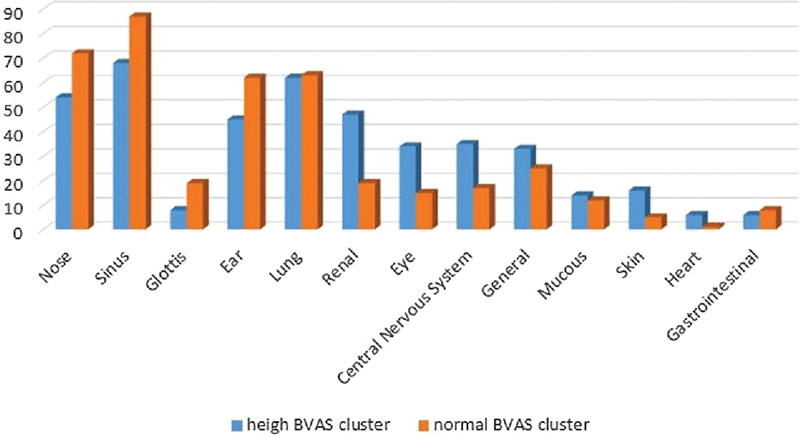
Result of the k-means.

Fig. 2 describes the first import data using excel data. Then select the attributes operator used in the model to select some attribute that needs clustering. All necessary operators are stored in this model. In this researcher used 164 patients of sample data on WG patients with 14 attributes. Based on this design, RapidMiner tools will classify the value of BVAS on the clusters that have been made. The final clustering results can be seen in the image below:

Figure 3 shows the result of the clustering with RapidMiner tools: cluster 0 was composed of 69 patients, and cluster 1, of 95 patients. High BVAS was considered with scores from 17 to 41, and normal BVAS was characterized by scores from 3 to 16 ( Table 3 and Fig. 4 and 5 ).

**Table 3:** Result of the k-means

Site of the clinical symptoms	High BVAS cluster	Normal BVAS cluster	N
Nose	54	72	126
Sinus	68	87	155
Glottis	8	19	27
Ear	45	62	107
Lung	62	63	125
Kidney	47	19	66
Eye	34	15	49
Central nervous Ssystem	35	17	52
General	33	25	58
Mucous membrane	14	12	26
Skin	16	5	21
Heart	6	1	7
Stomach and intestine	6	8	14

Abbreviation: BVAS, Birmingham Vasculitis Activity Score.

**Fig. 6:**
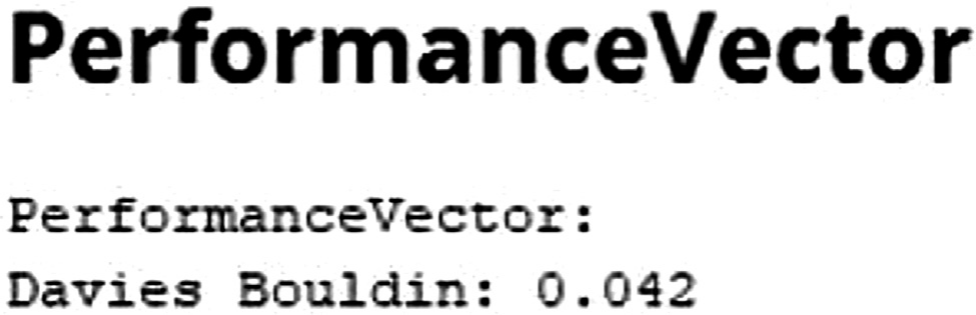
Performance.

One of the operators used to measure K-means performance. The performance measurement parameters are avg._within_centroid_distance and Davies Bouldin. The assessment parameter is an algorithm that produces clusters with low intra-cluster distance and high inter-cluster spacing will have a low. The Davies-Bouldin index (DBI) was used to evaluate the goodness of split by a K-Means clustering algorithm. The lower the DBI value, the better the clustering result. 12 The DBI of the WG patients was of 0.042 ( Fig. 6 ).

### Association of Ethnicity and Gender with Clinical symptoms

**Fig. 7:**
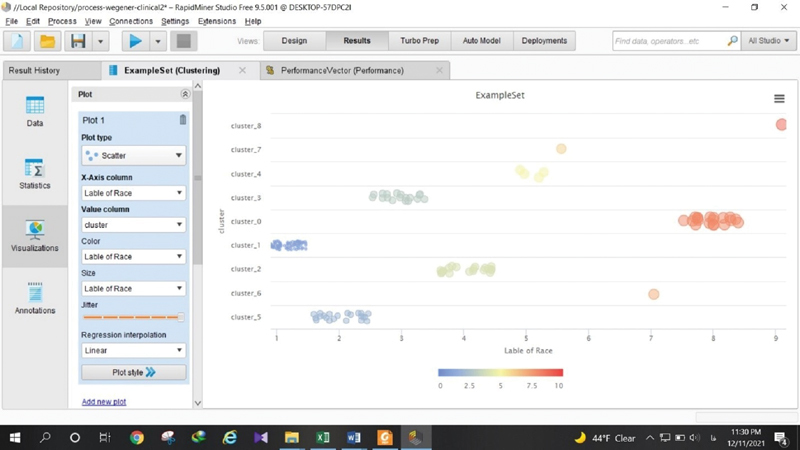
Output of the process.

The ethnicities of the 164 patients enrolled were Persian, Azari, Kurd, Lor, Arab, Gilaki, Baloch, Bakhtiari, and Turkman; 79 (42.47%) patients were male, and 107 (57.53%), female ( Table 4 ). The result of clustering has 9 clusters ( Fig. 7 and Table 5 ).

**Table 4:** Frequency distribution of the different ethnicities in the study

Ethnic groups	Total	verage a	Gender		Average BVAS
			Male	Female	
Persian	79	50	38	41	15.4
Azari	22	51	10	12	15.8
Gilaki	19	48	8	11	17.1
Kurd	19	54	7	12	16.3
Lur	18	55	7	11	14.7
Others	7	62	1	6	15.7
Total	164	51	79	107	15.4

Abbreviation: BVAS, Birmingham Vasculitis Activity Score.

**Table 5:** Results of the clustering process in RapidMiner

Name of cluster	N	Label of ethnicity	Name of ethnicity
Cluster0	19	8	Gilaki
Cluster1	79	1	Persian
Cluster2	19	4	Kurd
Cluster3	18	3	lor
Cluster4	4	5	Arab
Cluster5	22	2	Azari
Cluster6	1	7	Turkman
Cluster7	1	6	Bakhtiari
Cluster8	1	9	Balouch
Total	164		

### Persian Ethnicity

**Fig. 8:**
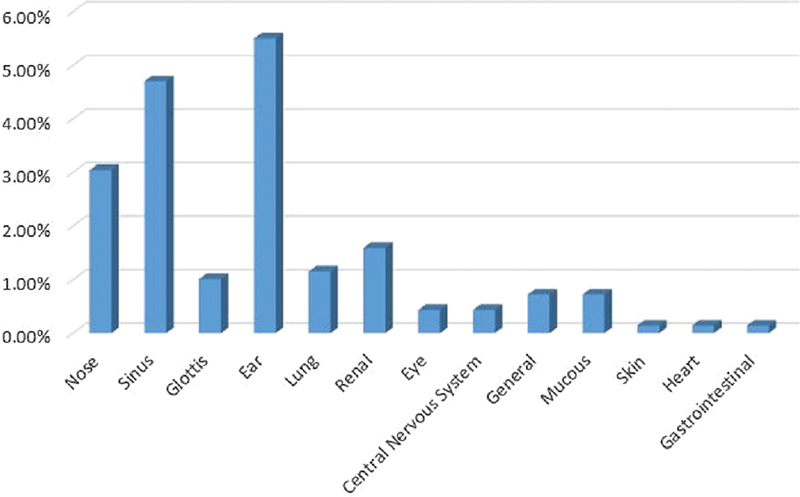
Persian Ethnicity.

Most patients were of Persian ethnicity (48.17%; female patients: 51.89%; male patients: 48.1%), with an average of age 50 years, and with the ears, sinus and nose respectively as the sites most frequently involved ( Table 6 and Fig. 8 ).

**Table 6:** Relative frequency of the site of the clinical symptoms among the study sample

Site of the clinical symptoms	N	Relative frequency
Ear	38	5.5%
Sinus	33	4.7%
Nose	21	3.04%
Kidney	11	1.59%
Lung	8	1.15%
Glottis	7	1.01%
General	5	0.72%
Mucous membrane	5	0.72%
Eye	3	0.43%
Central nervous system	3	0.43%
Heart	1	0.14%
Stomach and intestine	1	0.14%
Skin	1	0.14%

#### Azari Ethnicity

**Fig. 9:**
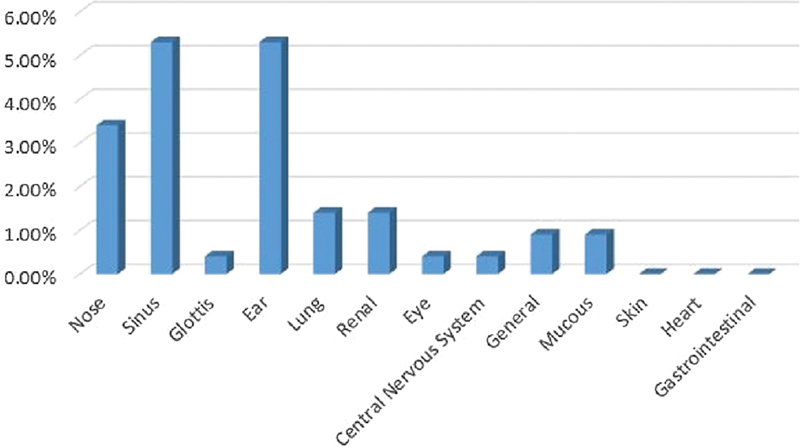
Azari Ethnicity.

The Azari ethnicity was the second highest (13.41%; female patients: 54.54%; male patients: 45.46%), and these patients had an average age of 51 years, and the ears, sinus, and nose were the most involved sites respectively ( Table 7 and Fig. 9 ).

**Table 7:** Relative frequency of the site of the clinical symptoms in patients of Azari ethnicity

Site of the clinical symptoms	N	Relative frequency
Ear	11	5.3%
Sinus	11	5.3%
Nose	7	3.4%
Kidney	3	1.4%
Lung	3	1.4%
General	2	0.9%
Mucous membrane	2	0.9%
Eye	1	0.4%
Glottis	1	0.4%
Central nervous system	1	0.4%
Skin	0	0%
Heart	0	0%
Stomach and intestine	0	0%

#### Kurd Ethnicity

**Fig. 10:**
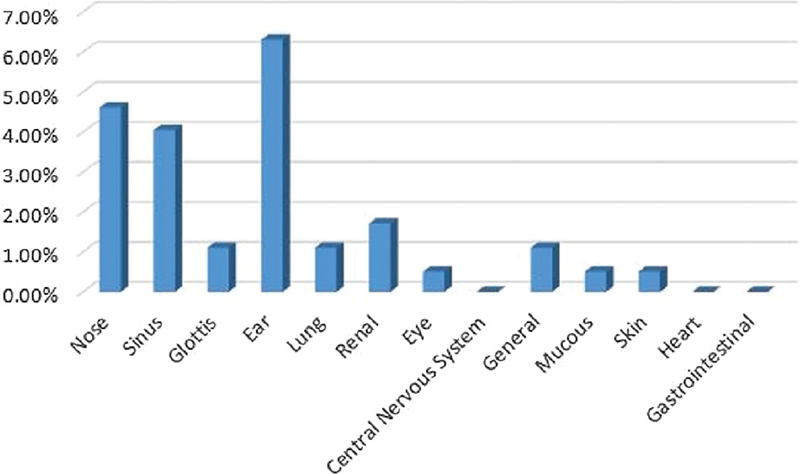
Kurd Ethnicity.

The rate of Kurd patients was of 11.17% (female patients: 63.15%; male patients: 36.85%); their average age was of 54 years and the ears, nose, and sinus were the most involved sites respectively ( Table 8 and Fig. 10 ).

**Table 8:** Relative frequency of the site of the clinical symptoms in patients of Kurd ethnicity

Site of the clinical symptoms	N	Relative frequency
Ear	11	6.3%
Nose	8	4.6%
Sinus	7	4.04%
Kidney	3	1.7%
General	2	1.1%
Lung	2	1.1%
Glottis	1	1.1%
Eye	1	0.5%
Mucous membrane	1	0.5%
Skin	1	0.5%
Central nervous system	0	0
Heart	0	0
Stomach and intestine	0	0

#### Gilaki Ethnicity

**Fig. 11:**
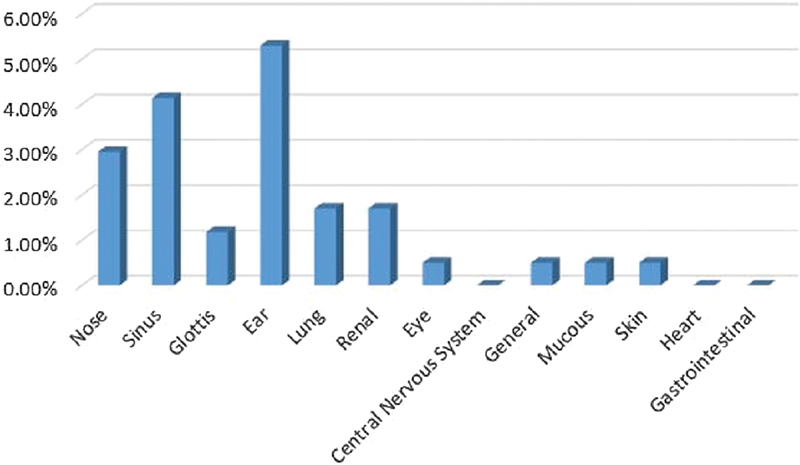
Gilaki Ethnicity.

The rate of Gilaki patients was of 11.17% (female patients: 57.89%; male patients: 42.11%); their average age was of 48 years and the ears, sinus, and nose were the most involved sites respectively ( Table 9 and Fig. 11 ).

**Table 9:** Relative frequency of the site of the clinical symptoms in patients of Gilaki ethnicity

Site of the clinical symptoms	N	Relative frequency
Ear	9	5.3%
Sinus	7	4.14%
Nose	5	2.95%
Kidney	3	1.7%
Lung	3	1.7%
Glottis	2	1.18%
Eye	1	0.5%
General	0	0.5%
Mucous membrane	1	0.5%
Skin	1	0.5%
Heart	0	0
Stomach and intestine	0	0
Central nervous system	0	0

#### Lor Ethnicity

**Fig. 12:**
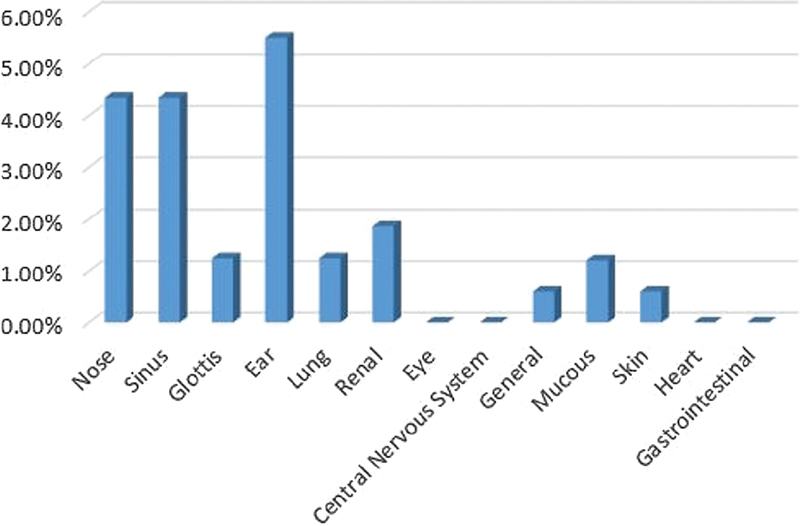
Lor Ethnicity.

The rate of Lor patients was of 10.9% (female patients: 61.1%; male patients: 38.9%); their average age was of 54 years and the ears, sinus, and nose were the most involved sites respectively ( Table 10 and Fig. 12 ).

**Table 10:** Relative frequency of the site of the clinical symptoms in patients of Lor ethnicity

Site of the clinical symptoms	N	Relative frequency
Ear	9	5.5%
Sinus	7	4.34%
Nose	7	4.34%
Kidney	3	1.86%
Lung	2	1.24%
Glottis	2	1.24%
Mucous membrane	2	1.2%
General	1	0.6%
Skin	1	0.6%
Heart	0	0
Stomach and intestine	0	0
Eye	0	0
Central nervous system	0	0

#### Other Ethnicities

There were few patients of the Bakhtiari, Arab, and Baluch ethnic groups, so they are not mentioned in this section.

**Fig. 13:**
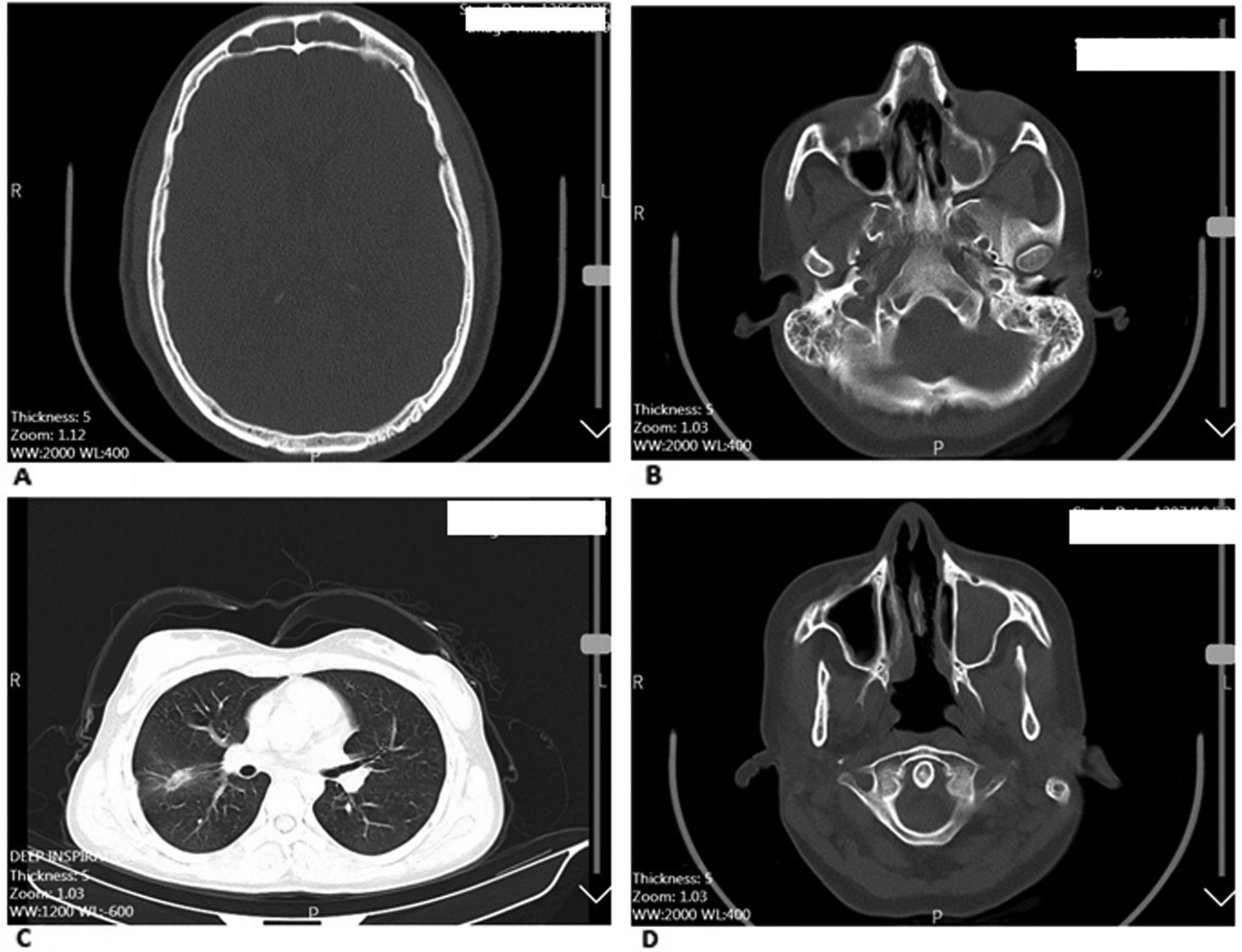
Radiographs of a Wegener granulomatosis patient: ( **A** ) frontal sinusitis; ( **B** ) bilateral mastoiditis and maxillary sinusitis; ( **C** ) lung infiltration; ( **D** ) perforation of the nasal septum and maxillary sinusitis.

**Fig. 14:**
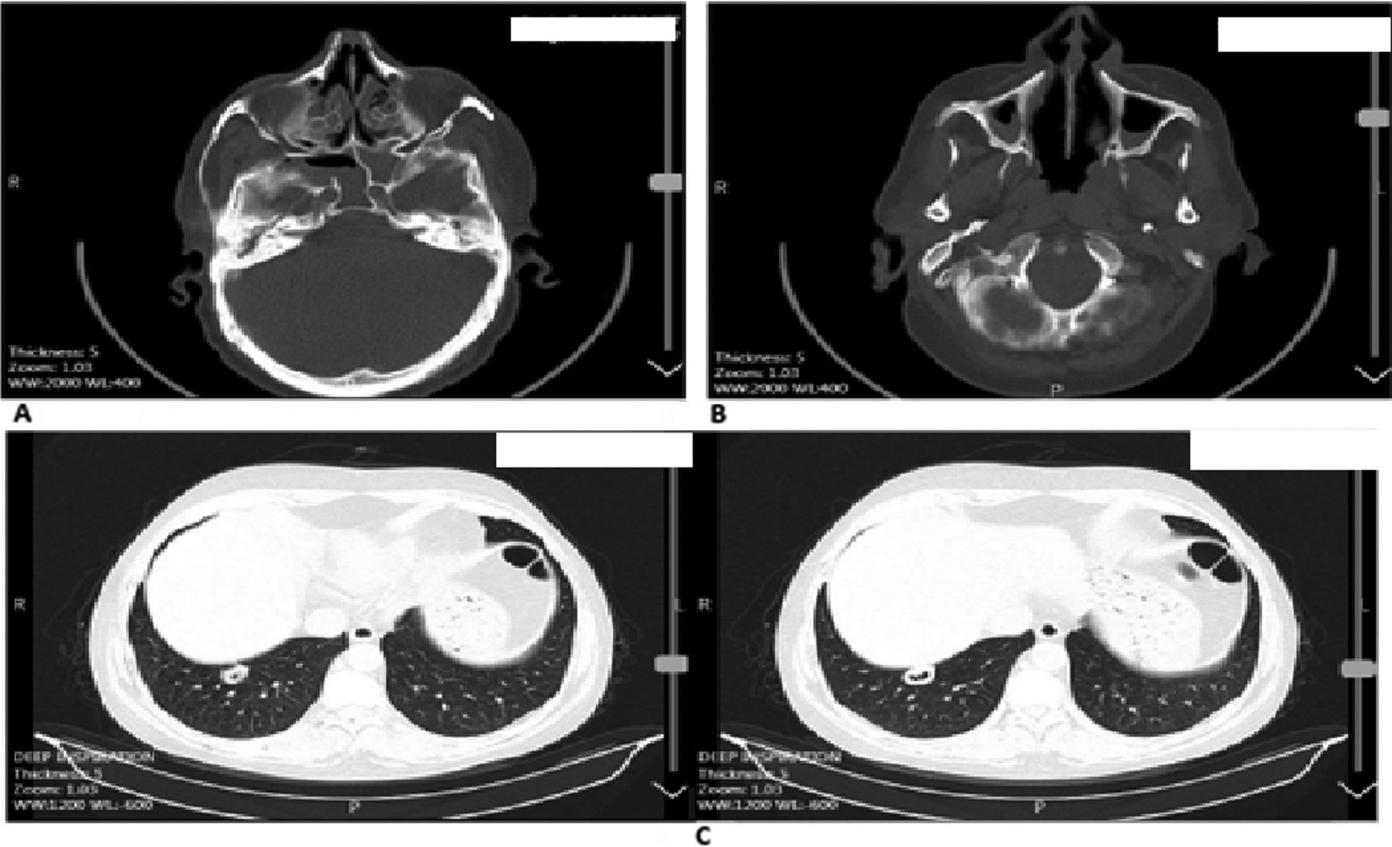
Radiographs of a Wegener granulomatosis patient: ( **A** ) ethmoidal sinusitis; ( **B** ) bilateral maxillary sinusitis; ( **C** ) lung cavity nodule.

Regarding all ethnic groups analyzed, the most common radiological findings were lung involvement, nodule or cavity formation, and infiltration secondary to GPA at diagnosis. In addition, another common finding, which was observed after a pneumologist reanalyzed the patients' lungs, was subsegmental atelectasis. The most common radiological findings related to sinus involvement were the presence of sinusitis and mastoid patients. Figures 13 and 14 show the radiographs of two WG patients.

## Discussion

The present study is the first analysis of the ethnicity of WG patients in Iran. Using geographic clustering and the data on the clinical symptoms of the study sample, we identified that the sites most involved were the ears, sinus, and nose for every ethnicity, but with a different order of most to least affected for each ethnic group.

In a similar study 14 conducted in Mumbai, India, the clinical manifestations, treatment, and outcomes of 42 patients (21 male and 21 female patients) from a single-center, tertiary care hospital were analyzed. Their median age was of 48.5 years. Pulmonary manifestations as well as renal, ear, nose, and throat manifestations were the most common upon presentation. Cutaneous and nervous system involvement were less common. The median BVAS was of 14.5. 14 The present study involved 164 (79 male and 85 female) patients with a median age of 51 years. Sinus and nose, lung, and ear involvement were the most common, and gastrointestinal and cardiac involvement were less common. The median BVAS was of 15.4. Therefore, the results show that the average age and BVAS of the patients in the present study were higher than those of the patients in the Indian study 14 ( *p* -value) and the number of female mitral is higher than that of men.

In this study, we intend to analyze respiratory and renal involvement. 40.2% of patients had Renal involvement. Renal involvement in Caucasians was 70% to 80%; in South India, 70.5% was reported. In our study renal involvement was less common. Upper and lower respiratory involvement was noted in 125 (76.2%) patients. Chronic sinusitis in 125 patients was the most common symptom, followed by Saddle nose in 42 patients. The other symptoms included hemoptysis in 28, subglottic stenosis in 22, and chronic sinusitis in 4 (11%) patients. Diffuse alveolar hemorrhage was seen in 3 (5%), and the other symptoms were not noted in any. Upper and lower respiratory involvement were much than the series from western and Asian data (76.2%). 15

In another study, 2 the results showed that the average age at diagnosis was between 20 and 40 years, that males are more affected than females, and that the initial manifestations were in the ear, nose and throat, lung, skin, and kidney. In the present study, the average age at diagnosis was of 42 years, and female patients were more affected than male subjects. Sinus, nose, lung, and ear involvement were more common, and the present study also revealed for the first time that the left ear was the first to show signs in all patients with ear involvement. This helps in the diagnosis of the disease, because, if the patient presents involvement of the left ear, we can diagnose WG more quickly. 2

Among the 164 patients of the present study, 48.17% were Persian, with a median BVAS of 15.4, and with the ears, sinus and nose as the most involved sites; 13.41% were Azari, with a median BVAS of 15.8, and the ears, sinus, and nose as the most involved sites; 11.17% were Kurds, with a median BVAS of 16.3, and the ears, nose, and sinus as the most involved sites. 11.17% were Gilaki, with a median BVAS of 17.1, and the ears, sinus, and nose as the most involved sites; and 10.9% were Lor, with a median BVAS of 14.7, and the ears, sinus, and nose as he most involved sites. In the present study, the clinical manifestations among the different Iranian ethnic groups were not different. But a similar study 16 conducted in France indicated different clinical presentations in white Europeans and subshrubs and Afro-Caribbeans, with black patients presenting severe granulomatous manifestations more frequently. And also expected that ethnicities in cold regions have a higher rate of patient involvement and high BVAS. But among the ethnic groups in the present study, the Gilaki had the highest BVAS because it has a humid climate, so, in Iran, in areas with humidity, the rate of the disease may be higher.

The limitation of the present study was the lack of examination of smokers among the patients. Data on smoking history was not available for all patients, and some patients did not provide any history of exposure to smoking. Therefore, we could not investigate the relationship between the disease and smoking, and due to the apparently small number of smokers, we were unable to compare smokers and non-smokers. Therefore, whether smoking may be related to the development and exacerbation of clinical symptoms is a hypothesis that requires a comparative study with many patients and comprehensive information on the history of exposure to smoking.

## Conclusion

The findings of the present study indicated that the most involved sites in WG were the sinus (causing sinusitis and mastoid, the nose (causing nasal ulcer and epistaxis), the and ears (causing conductive hearing loss); gastrointestinal and cardiac involvement were less common. However, involvement of the upper and lower respiratory tract was higher than that reported in Western and Asian case series. The present study also revealed for the first time that the left ear was the first to show signs in all patients with ear involvement. This helps in the diagnosis of the disease, because, if the patient presents involvement of the left ear, we can diagnose WG more quickly. Moreover, among the ethnic groups in the present study, the Gilaki had the highest BVAS because it has a humid climate, so, in Iran, in areas with humidity, the rate of the disease may be higher.
